# Features of digital signal processing algorithms using Galois fields GF(2^n^+1)

**DOI:** 10.1371/journal.pone.0293294

**Published:** 2023-10-25

**Authors:** Ibragim E. Suleimenov, Yelizaveta S. Vitulyova, Dinara K. Matrassulova

**Affiliations:** 1 National Engineering Academy of the Republic of Kazakhstan, Almaty, Kazakhstan; 2 Almaty University of Power Engineering and Telecommunications Named After Gumarbek Daukeyev, Almaty, Republic of Kazakhstan; Roma Tre University: Universita degli Studi Roma Tre, ITALY

## Abstract

An alternating representation of integers in binary form is proposed, in which the numbers -1 and +1 are used instead of zeros and ones. It is shown that such a representation creates considerable convenience for multiplication numbers modulo p = 2^n^+1. For such numbers, it is possible to implement a multiplication algorithm modulo p, similar to the multiplication algorithm modulo the Mersenne number. It is shown that for such numbers a simple algorithm for digital logarithm calculations may be proposed. This algorithm allows, among other things, to reduce the multiplication operation modulo a prime number p = 2^n^+1 to an addition operation.

## Introduction

Currently, non-binary Galois fields are increasingly used in information technologies [1, 2], in particular, in information security systems [3, 4]. The example is a variety of Galois fields *GF*(*p*), which are residue classes of the ring of integers modulo some prime number *p*. The advantages of using such fields for digital signal processing were clearly demonstrated in [5, 6].

There is also a number of reports devoted to the development of electronic circuits that perform addition and multiplication operations modulo in current literature, for example, [7, 8]. Such reports are closely related to research in the field of practical use of Galois fields, since the operations of addition and multiplication modulo an integer can be considered as operations on the elements of the Galois field. The interest is connected, among other things, with the fact that such operations are of significant interest for cryptography [9, 10].

The development of electronic circuits operating in Galois fields is also of interest from the point of view of improving artificial intelligence (AI) systems. Namely, as it was shown in the reports [11, 12] the further development of AI, assuming its gradual approach to the biological prototype, cannot exclude the transition to multivalued logic, since human thinking is irreducible to binary logic. It is appropriate to emphasize that the problem of creating of AI approaching a biological prototype is of considerable interest, including from the point of view of revealing the essence of intelligence as such [13, 14].

Multivalued logic, dating back to the works of Jan Lukasiewicz [15], has been actively developing recently [16–19], but the level of its practical use obviously does not meet the existing potential [11]. If the number of values accepted by variables of multivalued logic is equal to an integer power of some prime number, these values can be put in one-to-one correspondence to the elements of the Galois field. Consequently, any operations in this case are reduced to addition and multiplication, i.e., the improvement of electronic circuits that perform addition and multiplication modulo an integer is also of interest from this point of view. The possibilities arising in this case are disclosed, in particular, in [20].

In turn, among the simple Galois fields *GF*(*p*), a special place is occupied by fields for which the number *p* is equal to the Mersenne prime number. Such numbers are representable as

$$p=2^{n}-1$$ (1)

where *n* are specifically selectable integers, the first of which are, 2, 3, 5, and 7.

Such numbers are used, in particular, to generate pseudorandom numbers [19]. Specifically, a pseudorandom number generator called the Mersenne twister is known, developed in 1997 by Japanese scientists M. Matsumoto and T. Nishimura [21], directly based on the use of Mersenne numbers.

There are works in which the expediency of using such numbers for data transmission is justified [21, 22], etc.

A very remarkable feature of the Mersenne numbers *p*_*m*_ is the fact that Galois fields *GF*(*p*_*m*_) allows one to implement fairly simple electronic circuits that perform addition and multiplication operations modulo *p*_*m*_, i.e., those operations to which any other operations performed on the elements of the field *GF*(*p*_*m*_) are reduced. Examples of such systems and algorithms of their functioning are reflected in the current literature, for example [23].

In this paper, it is shown that Galois fields, *GF*(*p*), for which *p* = 2^*n*^+1., are also of considerable interest for digital signal processing. One of these fields is the *GF*(257) field, which is advisable to use for digital signal processing with a standard number of levels equal to 256. Thus, it can be argued that for this case there is a very specific Galois field, which, among other things, allows you to bring signals that meet existing standards [24], to logical operations.

## Advantages of Mersenne numbers for computing systems modulo an integer

In relation to digital signal processing, the following property of Mersenne numbers is of interest. Multiplication of a number *a* written in binary form by 2 modulo the Mersenne number is recused to cyclic permutation of characters takes place. For example, for calculations in the field *GF*(7), next equality is true

$$2∙a_{2}a_{1}a_{0}{=}_{\left(7\right)}a_{1}a_{0}a_{2}$$ (2)

where *a*_*i*_ are binary characters.

The convenience of using such a property for digital signal processing in Galois fields is as follows. Let us consider the field *GF*(127) whose characteristic is a Mersenne number with *n* = 7. Note that this example is also important from a practical point of view, since a scale with 127 levels is often used in digital signal processing too. In binary form, any of the elements of the *GF*(127) field can be represented as

$$A=2^{6}∙a_{6}+2^{5}∙a_{5}+\cdots +2^{1}∙a_{1}+2^{0}∙a_{0}$$ (3)

We emphasize that the number of elements represented in the form (3) when calculating in terms of ordinary integers is 128, but

1111111≡0000000(127) (4)

Consequently, the number of field elements given by Formula (3) is indeed 127.

Let the element *A*, represented in the form (3), be multiplied modulo 127 by the element *B*, represented in the same form.


$$B=2^{6}∙b_{6}+2^{5}∙b_{5}+\cdots +2^{1}∙b_{1}+2^{0}∙b_{0}$$ (5)

Then the result of the product is the sum of the following terms

$$A∙b_{0}=(2^{6}∙a_{6}+2^{5}∙a_{5}+\cdots +2^{1}∙a_{1}+2^{0}∙a_{0})b_{0}$$

$$2^{1}∙A∙b_{1}=(2^{6}∙a_{5}+\cdots +2^{2}∙a_{1}+2^{1}∙a_{0}+2^{0}∙a_{6})b_{1}$$ (6)

⋯

$$2^{6}∙A∙b_{6}=(2^{6}∙a_{0}+2^{5}∙a_{6}+\cdots +2^{1}∙a_{2}+2^{0}∙a_{1})b_{6}$$

Grouping the terms at the same powers of two, we get

$$c_{6}=a_{6}b_{0}+a_{5}b_{1}+\cdots +a_{0}b_{6}$$

$$c_{5}=a_{5}b_{0}+a_{4}b_{1}+\cdots +a_{6}b_{6}$$ (7)

⋯

$$c_{0}=a_{0}b_{0}+a_{6}b_{1}+\cdots +a_{1}b_{6}$$

We emphasize that although Formula (7) include only quantities taking the values 0 or 1, no such restrictions are imposed on the summation result itself, i.e., the sum is calculated in the sense of the original field *GF*(127).

The algorithm based on Formula (7) allows for a fairly simple circuit implementation, therefore, it makes sense to consider whether there is no way to implement its analogue for fields *GF*(2^*n*^+1), in particular, for the field *GF*(257). This is due to an obvious consideration: the signal digitization scale, which provides for the use of 256 levels, is one of the most common [24].

## Algorithms of calculations modulo in the special case of the field *GF*(17)

Let’s start from the consideration of one of the simplest fields of type *GF*(2^*n*^+1), specifically from the field *GF*(17).

This field, as well as other Galois fields, is a ring of residue classes of the ring of integers, in this case modulo 17. Traditionally, positive integers are used when representing field elements, however, this is not mandatory at all. In particular, as emphasized in [25], it is advisable to use a set of elements {−1,0,1} to represent the field *GF*(3), where the use of curly brackets emphasizes that the set is being considered.

Similarly, the field *GF*(17) can be considered as a set of elements

GF(17)={−8,−7,…,0,…7,8} (8)

The selection of the representing elements is arbitrary up to the modulo comparison operation, for example,

−8≡9(17) (9)

The advantages of such a choice for the purposes of this work are demonstrated by Tables 1 and 2. In these tables, the degrees of the elements of the field under consideration are counted in the usual representation through positive integers and in the representation (8), respectively.

**Table 1:** The degrees of the elements of the *GF*(17) field in terms of positive integers.

*n* = 0	*n* = 2	*n* = 4	*n* = 8	*n* = 16
1	1	1	1	1
2	4	16	1	1
3	9	13	16	1
4	16	1	1	1
5	8	13	16	1
6	2	4	16	1
7	15	4	16	1
8	13	16	1	1
9	13	16	1	1
10	15	4	16	1
11	2	4	16	1
12	8	13	16	1
13	16	1	1	1
14	9	13	16	1
15	4	16	1	1
16	1	1	1	1

**Table 2:** Degrees of elements of the field *GF*(17) in the representation (8).

*n* = 0	*n* = 2	*n* = 4	*n* = 8	*n* = 16
1	1	1	1	1
2	4	-1	1	1
3	-8	-4	-1	1
4	-1	1	1	1
5	8	-4	-1	1
6	2	4	-1	1
7	-2	4	-1	1
8	-4	-1	1	1
-8	-4	-1	1	1
-7	-2	4	-1	1
-6	2	4	-1	1
-5	8	-4	-1	1
-4	-1	1	1	1
-3	-8	-4	-1	1
-2	4	-1	1	1
-1	1	1	1	1

Both tables demonstrate the fact that all nonzero elements of the field under consideration, as follows from the general theory of Galois fields, obey the equation

$$x^{16}-1=0$$ (10)

From this relation, in particular, it follows that any element of the field under consideration can be represented as

$$x={{\left(\sqrt[{2}]{1}\right)}_{4}}^{s_{4}}{{\left(\sqrt[{4}]{1}\right)}_{3}}^{s_{3}}{{\left(\sqrt[{8}]{1}\right)}_{2}}^{s_{2}}{{\left(\sqrt[{16}]{1}\right)}_{1}}^{s_{1}}$$ (11)

where *s*_*i*_ = 0; 1.

We show this by first revealing the meaning of the formal notation ${\left(\sqrt[{k_{2}}]{1}\right)}_{k_{1}}$ using Tables 1 and 2.

The ratio (10) can be rewritten in the form

$${\left(x^{8}\right)}^{2}-1=0$$ (12)

Formula (12) emphasizes that there are only two different values of the element *z* = *x*^8^. This corresponds to the fact that in the fourth column of Tables 1 and 2 there are only two different elements.

There are two other similar forms of representation of the relation (10).


$${\left(x^{4}\right)}^{4}-1=0$$ (13)

$${\left(x^{2}\right)}^{8}-1=0$$ (14)

Formula (13) emphasizes that there are four different values of the element *z* = *x*^4^, which are found in the third columns of Tables 1 and 2. Similarly, as the ratio (14) shows, there are eight different elements *z* = *x*^2^, which are found in the second columns of these tables.

Accordingly, formally we can write

$${\left(\sqrt[{16}]{1}\right)}_{1}=16$$ (15)

$${\left(\sqrt[{16}]{1}\right)}_{2}=4;13$$ (16)

$${\left(\sqrt[{16}]{1}\right)}_{3}=2;8;9;15$$ (17)

The eight remaining y elements highlighted in Tables 1 and 2 in color represent primitive elements. They have the property that *y*^*m*^ = 1 if and only if *m* = 16. We have

$${\left(\sqrt[{16}]{1}\right)}_{4}=3;5;6;7;10;11;12;14$$ (18)

The proved relation (11) follows directly from Formulas (15)–(18), and the choice of elements ${\left(\sqrt[{k_{2}}]{1}\right)}_{k_{1}}$ is determined by the following considerations. Any nonzero element of the field under consideration represents some degree of one of the primitive elements *y* listed in the right part of Formula (18). This follows from the general theory of Galois fields, and for clarity it can be demonstrated as follows.

All the degrees of the primitive element y of the field under consideration from 0 to 15 are different. At the same time, all these degrees are the roots of Eq (10), i.e., they exhaust the elements of the field.

Consider the power of *y*^*m*^ and represent the number m, where 0≤*m*≤15 in binary form

$$m=m_{4}m_{3}m_{2}m_{1}$$ (19)

where *m*_*i*_ are binary characters.

Such an entry means that

$$m=2^{3}∙m_{4}+2^{2}∙m_{3}+2^{1}∙m_{2}+2^{0}∙m_{1}$$ (20)

Therefore, the degree *y*^*m*^ is representable as

$$y^{m}={\left(y^{8}\right)}^{m_{4}}{\left(y^{4}\right)}^{m_{3}}{\left(y^{2}\right)}^{m_{2}}{\left(y^{1}\right)}^{m_{1}}$$ (21)

This expression coincides with (11) if we put the corresponding root of unity equal to one of the powers of the primitive element appearing in (21).

For further it is essential that when moving to the representation of the elements of the field under consideration in the form (8), the roots of unity (15)–(18) acquire a symmetrical form that and shows Table 2.


$${\left(\sqrt[{16}]{1}\right)}_{1}=-1$$ (22)

$${\left(\sqrt[{16}]{1}\right)}_{2}=4;-4$$ (23)

$${\left(\sqrt[{16}]{1}\right)}_{3}=2;8;-8;-2$$ (24)

$${\left(\sqrt[{16}]{1}\right)}_{4}=3;5;6;7;-7;-6;-5;-3$$ (25)

This determines the convenience of representation in the form (8): all elements of the field under consideration, with the exception of primitive ones, are representable as powers of two, which is also emphasized by Table 2.

This fact generates another efficient algorithm for multiplying field elements, de facto based on the digital logarithm method. Digital logarithm has been considered in many reports, in particular in [26, 27], but this problem, if put in a general form, remains unresolved.

However, for practical needs, it can be limited to solving it for specific Galois fields. In particular, within the framework of this work, it is solved in relation to Galois fields of the form *GF*(2^*p*^+1), which, as noted above, also includes the field *GF*(257), which corresponds to the number of digital signal levels that is often used in practice.

In relation to the *GF*(17) field, the digital logarithm algorithm can be constructed as follows.

1. The field element is identified as belonging to a set of primitive elements.
2. If this element belongs to the specified set, then the value of *m*_1_ in Formula (21) is chosen to be 1, if not, then zero.
3. If *m*_1_ = 1, then the element in question is multiplied by 3 modulo 17. As a result of the representation in the form (8), the element is reduced to the power of two. When using binary notation, this means that the logical unit will stand only on one of the positions, which identifies the exponents *m*_2_, *m*_3_ and *m*_4_.
4. The set of exponents of degrees *m*_*i*_ completes the procedure of digital logarithm.

Solving the digital logarithm problem, in turn, makes it possible to significantly simplify the algorithm for multiplying field elements on each other. Indeed, in this case, the operation is reduced to the addition of numbers (20) modulo 16. Carrying out such an operation by circuit means does not cause difficulties, since it boils down to the usual addition of binary numbers with the rejection of the highest digit.

The described algorithm really makes it possible to significantly simplify the multiplication operation in the *GF*(17) field, but, firstly, the question of the circuit identification of primitive elements remains open, and secondly, this algorithm is very specific. Indeed, it is built on the fact that any of the primitive elements is reduced to the power of two (with a positive or negative sign) by multiplying by a fixed element (for example, by 3). This situation is not realized for other Galois fields of the type under consideration, in particular, for the field *GF*(257), which is of primary interest from the point of view of practical applications.

## Alternating binary encoding

We show that the indicated problem is solved using alternating encoding of elements of Galois fields of the type under consideration.

Again, let’s start from the example of the field *GF*(17). Consider an expression of the form (3), but only now we will understand by *a*_*i*_ the numbers taking the values +1 and -1.


$$A=2^{3}∙a_{3}+2^{2}∙a_{2}+2^{1}∙a_{1}+2^{0}∙a_{0}$$ (26)

It can be easily shown that the result of calculations by Formula (3) in this case will certainly give an odd number.

In total, there are 2^4^ such combinations of the form (3), and the maximum number is

$$A_{max}=2^{3}+2^{2}+2^{1}+2^{0}=15,$$ (27)

and, accordingly, *A*_*min*_ = −15.

Otherwise, the set of numbers given by Formula (26) coincides with the set of odd numbers in the range from -16 to +16. We exclude zero from consideration, which is quite justified, since the multiplication operation is being considered. Then the number of combinations of the form (26) in the case under consideration coincides with the number of non-zero elements of the field *GF*(17).

All possible combinations are listed in Tables 3 and 4. Table 3 refers to the elements of the field that are not primitive, Table 4 –vice versa. The first four columns of these tables display the numbers *a*_*i*_ appearing in expressions (26), the fifth column displays the result of summation in terms of ordinary integers *A*_1_, the sixth–displays the result of reduction modulo 17 to the form (8) A.

**Table 3:** Alternating binary representation of elements of the *GF*(17) field that are not primitive.

*a*_3_	*a*_2_	*a*_1_	*a*_0_	*A*_1_	*A*
1	-1	-1	-1	1	1
-1	-1	-1	-1	-15	2
-1	-1	-1	1	-13	4
-1	-1	1	1	-9	8
-1	1	1	1	-1	1
1	1	1	1	15	-2
1	1	1	-1	13	-4
1	1	-1	-1	9	-8
1	-1	-1	-1	1	1

**Table 4:** Alternating binary representation of primitive elements of the *GF*(17) field.

*a*_3_	*a*_2_	*a*_1_	*a*_0_	*A*_1_	*A*
1	-1	-1	1	3	3
-1	-1	1	-1	-11	6
-1	1	-1	1	-5	-5
1	-1	1	1	7	7
-1	1	1	-1	-3	-3
1	1	-1	1	11	-6
1	-1	1	-1	5	5
-1	1	-1	-1	-7	-7
1	-1	-1	1	3	3

It can be seen that the modulo reduction field of the numbers represented in the form (26) exhaust the set of nonzero elements of the field *GF*(17). Consequently, such a representation can be used along with any other, especially if we take into account that representatives of the residue classes of the ring of integers modulo a prime number can be chosen arbitrarily.

As applied to the field under consideration, a representation of the form (26) in which *a*_*i*_ = ±1 has a property similar to the property possessed by Mersenne primes. Namely, the multiplication of the number written in the representation (26), which hereafter we will call alternating, by two can be displayed as the following operation

$$2∙A=2^{3}∙a_{2}+2^{2}∙a_{1}+2^{1}∙a_{0}-2^{0}∙a_{3}$$ (28)

This follows from the fact that the element -1 is the root of Eq (12) considered at *z* = *x*^8^ or from the fact that in the field under consideration 2^4^≡−1(17).

Consequently, the operation of multiplication by two when using an alternating binary representation of a number is reduced to a cyclic permutation of binary elements in the entry resulting from (18) with a change in the sign of the element being rearranged. We have

$$2∙A=2∙a_{2}a_{1}a_{0}a_{3}=a_{2}a_{1}a_{0}(-a_{3})$$ (29)

It can be seen that this property is indeed analogous to the property possessed by the operation of multiplication by two in fields formed using Mersenne numbers (2).

Property (29), in particular, allows you to implement an algorithm for multiplying two elements of the field *GF*(17), represented in alternating binary form, similar to the algorithm given by Formulas (6), (7).

Indeed, the result of multiplying the number *A*, represented in alternating form (26), by the number *B*, represented in the same form

$$B=2^{3}∙b_{3}+2^{2}∙b_{1}+2^{1}∙b_{1}+2^{0}∙b_{0}$$ (30)

it is the sum of the following terms

$$A∙b_{0}=(2^{3}∙a_{3}+2^{2}∙a_{2}+2^{1}∙a_{1}+2^{0}∙a_{0})b_{0}$$ (31)

$$2^{1}∙A∙b_{1}=(2^{3}∙a_{2}+2^{2}∙a_{1}+2^{1}∙a_{0}-2^{0}∙a_{3})b_{1}$$ (32)

$$2^{2}∙A∙b_{2}=(2^{3}∙a_{1}+2^{2}∙a_{0}-2^{1}∙a_{3}-2^{0}∙a_{2})b_{2}$$ (33)

$$2^{3}∙A∙b_{3}=(2^{3}∙a_{0}-2^{2}∙a_{3}-2^{1}∙a_{2}-2^{0}∙a_{1})b_{3}$$ (34)

Grouping elements at powers of two, we obtain the following expressions for the coefficients *c*_*i*_, that arise when multiplying two elements of the field

$$c_{1}=a_{3}b_{0}+a_{2}b_{1}+a_{1}b_{2}+a_{0}b_{3}$$ (35)

$$c_{2}=a_{2}b_{0}+a_{1}b_{1}+a_{0}b_{2}-a_{3}b_{3}$$ (36)

$$c_{3}=a_{1}b_{0}+a_{0}b_{1}-a_{3}b_{2}-a_{2}b_{3}$$ (37)

$$c_{4}=a_{0}b_{0}-a_{3}b_{1}-a_{2}b_{2}-a_{1}b_{3}$$ (38)

It can be seen that in Formulas (35)–(38) the coefficients *a*_*i*_ are rearranged cyclically with a sign change, which corresponds to the specifics of the field under consideration. We also emphasize that the coefficients *c*_*i*_ are not necessarily equal to ±1, i.e., they are not coefficients in the representation of the form (26). These are the weights with which the powers of two are added when calculating the result of the product.

The algorithm based on Formulas (35)–(38) can be implemented quite simply schematically, moreover, it admits an obvious generalization to any fields of *GF*(2^*n*^+1). However, the fact that the coefficients *c*_*i*_ are not coefficients in binary alternating representation makes us consider further simplifying the circuit implementation of multipliers in fields of the type under consideration.

## Algorithmic basis of digital logarithm in the *GF*(2^*n*^+1) field

A significant simplification of the circuit implementation of the multiplier in the field under consideration can be achieved by using the digital logarithm operation, as noted above.

In relation to the field *GF*(17) under consideration, the operation of digital logarithm is reduced to the operation of circuit identification of primitive elements. As can be seen from Table 2, multiplication by a primitive element leads to the fact that the number becomes equal to the power of two, which is easily identified schematically when using binary representation.

To identify primitive elements, you can use the following technique, which follows from the property of a cyclic permutation with a change in the sign of the element being rearranged. In the Table 5 presents examples of continuation of the sequence of characters *a*_*i*_, appearing in the alternating binary representation of the elements of the field *GF*(17). Each of these sequences may include one additional element -*a*_3_, as illustrated in Table 5.

**Table 5:** Example of continuation of a sequence of binary characters with alternating representation of elements of the *GF*(17) field.

*a*_3_	*a*_2_	*a*_1_	*a*_0_	−*a*_3_
1	-1	-1	-1	-1
-1	-1	-1	-1	1
-1	-1	-1	1	1

Let’s form the function *Q*_*j*,*j*−1_, which provides counting the number of sign variables in sequences similar to those presented in Table 5. This function is defined as follows

$$q_{i}=Q_{j,j-1}=\left\{ \begin{array}{c} 1,a_{j}a_{j-1}=-1\\ 0,a_{j}a_{j-1}=+1 \end{array}\right.$$ (39)

The values of *q*_*i*_ values for non–primitive field elements are shown in Table 6, and for primitive elements—in Table 7.

**Table 6:** Values of *q*_*i*_ values and their sums for elements of the *GF*(17) field that are not primitive.

*q*_3_ = *Q*_3,2_	*q*_2_ = *Q*_2,1_	*q*_1_ = *Q*_1,0_	*q*_0_ = *Q*_0,3_	∑*q*_*i*_
1	0	0	0	1
0	0	0	1	1
0	0	1	0	1
0	1	0	0	1
1	0	0	0	1
0	0	0	1	1
0	0	1	0	1
0	1	0	0	1
1	0	0	0	1

**Table 7:** Values of *q*_*i*_ values and their sums for primitive elements of the *GF*(17) field.

*q*_3_ = *Q*_3,2_	*q*_2_ = *Q*_2,1_	*q*_1_ = *Q*_1,0_	*q*_0_ = *Q*_0,3_	∑*q*_*i*_
1	0	1	1	3
0	1	1	1	3
1	1	1	0	3
1	1	0	1	3
1	0	1	1	3
0	1	1	1	3
1	1	1	0	3
1	1	0	1	3

The table data show that the sums ∑*q*_*i*_ are invariants for the set of primitive elements of the field *GF*(17) (all of them, when using the alternating binary representation of the elements of this field, are formed by a cyclic permutation with a sign change), as well as for the set of elements that are not primitive. In one case, the invariant is 1, in the other– 3.

This approach, based on the calculation of invariants, can easily be generalized to other fields of *GF*(2^*n*^+1), which directly follows from the fact that multiplication by two reduces to a cyclic permutation with a sign change. In particular, the nonzero elements of the field *GF*(257) decompose into 16 subsets, each of which is formed by cyclic permutations of the type under consideration. This, in turn, follows from the fact that in the field *GF*(257) there is

$$2^{8}\equiv -1(257)$$ (40)

## Conclusions

Thus, along with a direct algorithm for multiplying numbers in alternating binary representation, given by Formulas (35)–(38), we can propose the following algorithm for performing the multiplication operation for fields *GF*(2^*n*^+1), in particular, for the field *GF*(17).

1. The multiplied numbers are translated into binary alternating representation. This is provided by direct recalculation using the formula *A* = 2*A*_0_+1, where *A*_0_ is the original number, followed by the representation of the resulting odd number in the form (26).
2. Using binary alternating coefficients of the number *A* = 2*A*_0_+1, the invariants ∑*q*_*i*_ are calculated, which determine the division of the field into subsets, each of which corresponds to multiplication by a power of two.
3. Multiplication is performed by the element corresponding to the invariant ∑*q*_*i*_. As a result, an element is formed that represents the power of two (taking into account the sign). This degree sets the digital logarithm of the field element in question.
4. The exponents are added modulo the power of two, which schematically corresponds to the usual operation of adding binary numbers with dropping the highest digit.
5. The reverse transition from the exponent to the non-zero element of the field *GF*(2^*n*^+1) is carried out.

In general, the paper shows that the use of alternating binary representation for elements of fields *GF*(2^*n*^+1) allows them to realize all the same advantages that occur when working with Galois fields corresponding to Mersenne primes.

The most important type of fields of this type is the *GF*(257) field, since it corresponds to the number of levels of the digitized signal, which is often used in practice. For example, the most commonly used analog-to-digital converters assume the use of 256 levels.
